# Chronic hepatitis B complicated with secondary hemochromatosis was cured clinically: A case report

**DOI:** 10.1515/med-2023-0693

**Published:** 2023-03-30

**Authors:** Yun Ye, Jing Xie, Lina Wang, Cong He, Youwen Tan

**Affiliations:** Department of Hepatology, The Third Hospital of Zhenjiang Affiliated Jiangsu University, Zhenjiang 212003, Jiangsu Province, China

**Keywords:** chronic hepatitis B, hemochromatosis, clinical cure, case report

## Abstract

Chronic hepatitis B (CHB) often causes iron overload in the liver but rarely causes severe secondary hemochromatosis (SH). A 48-year-old man was infected with CHB via vertical transmission. For 21 years, nonstandard treatment with second-line hepatitis B antiviral drugs has been administered. Repeated abnormalities in the liver transaminase function and continuous low-level replication of the hepatitis B virus (HBV) have been detected. The skin had turned black 5 years back. Biochemical tests and imaging revealed the presence of hemochromatosis. A liver biopsy suggested severe iron overload. Two genetic tests ruled out hereditary hemochromatosis. The patient was diagnosed with SH and treated with 400 ml bloodletting once per week and an iron-chelating agent. After 12 weeks, liver function was normal, and the skin turned white. First, hepatitis B surface antigen (HBsAg) was lost, and HBV DNA was copied at low levels. The patient was diagnosed with an occult hepatitis B infection. HBV DNA was undetectable after 4 weeks of antiviral treatment with tenofovir. Upon reviewing the patient’s medical history, hemochromatosis was believed to be related to CHB with chronic inflammatory damage and no complete virological response. Improvements in hemochromatosis may promote HBsAg disappearance.

## 1 Introduction

Hereditary hemochromatosis (HH), also known as primary hemochromatosis, is an autosomal, recessive genetic disease. Iron overload and deposition in the liver, pancreas, heart, joints, skin, and reproductive system result in tissue and organ damage, mainly manifesting as cirrhosis, diabetes, arthralgia, and skin pigmentation [1,2]. The main disease of primary hemochromatosis is caused by a mutation in the hemochromatosis gene (HFE gene, i.e., typical HH). Studies have reported that the incidence is approximately 1.5/1,000–3.0/1,000 people worldwide and approximately 1/220–1/250 in the Caucasian population [3], with a male/female sex ratio of 3:1. Hemochromatosis is more common in patients aged 40–60 years old [4,5].

However, HH is relatively rare in China. Compared with Western countries, the genetic variation in primary hemochromatosis in China (e.g., yellow and Han races) is significantly different from that in Caucasians, and most cases are nonclassical HH caused by non-HEF gene variations [6,7]. Secondary hemochromatosis (SH) is commonly observed in patients with anemia caused by iron utilization disorders, hemolytic anemia, multiple massive blood transfusions, and excessive iron intake. The liver is the earliest and most severely affected iron deposition site. Iron deposition in the liver is often associated with the severity of liver disease in patients with chronic hepatitis B (CHB) [8]. Severe hemochromatosis secondary to CHB infection is rare. Herein, we report a case of hemochromatosis, secondary to CHB 21 years later that was clinically cured, and the loss of hepatitis B surface antigen (HBsAg) was found to be related to hemochromatosis.

## 2 Case presentation

A 48-year-old man was diagnosed with hepatitis B virus (HBV) infection 21 years before and had a family history of hepatitis B infection (his mother and sister had chronic HBV infections). The patient’s condition was divided into four stages. The first stage, from 2003 to 2010, involved the use of second-line antiviral drugs. Regular inspection revealed that alanine aminotransferase (ALT) and aspartate aminotransferase (AST) levels were elevated in the liver and HBV DNA was >10^4^ IU/L; the patient was diagnosed with CHB by a doctor. He began receiving lamivudine antiviral treatment in 2003. One year later, liver function was normal, and HBV DNA was negative (<500 IU/ml); in 2009, ALT and AST levels of the liver function were abnormal, and HBV DNA rebounded to 10^4^ IU/ml. Drug resistance testing revealed that the rtM204V/I/S site was resistant, and the patient was treated with lamivudine and adefovir dipivoxil. In 2010, the HBV DNA levels were below the lower detection limit (<500 IU/ml). However, the liver function ALT levels remained 1–2 times the upper limit of the normal (ULN), and alkaline phosphatase (ALP) and glutamine aminotransferase levels remained normal at this stage.

The second stage, from 2011 to 2018, involved traditional Chinese medicine treatment. During this period, the patient had HBV DNA replication at a low level (10^2^–10^4^) and had no special symptoms; however, the ALT and AST levels were still repeatedly abnormal. Therefore, antiviral treatment was discontinued and he was switched to traditional Chinese medicine (details unknown).

**Figure 1:**
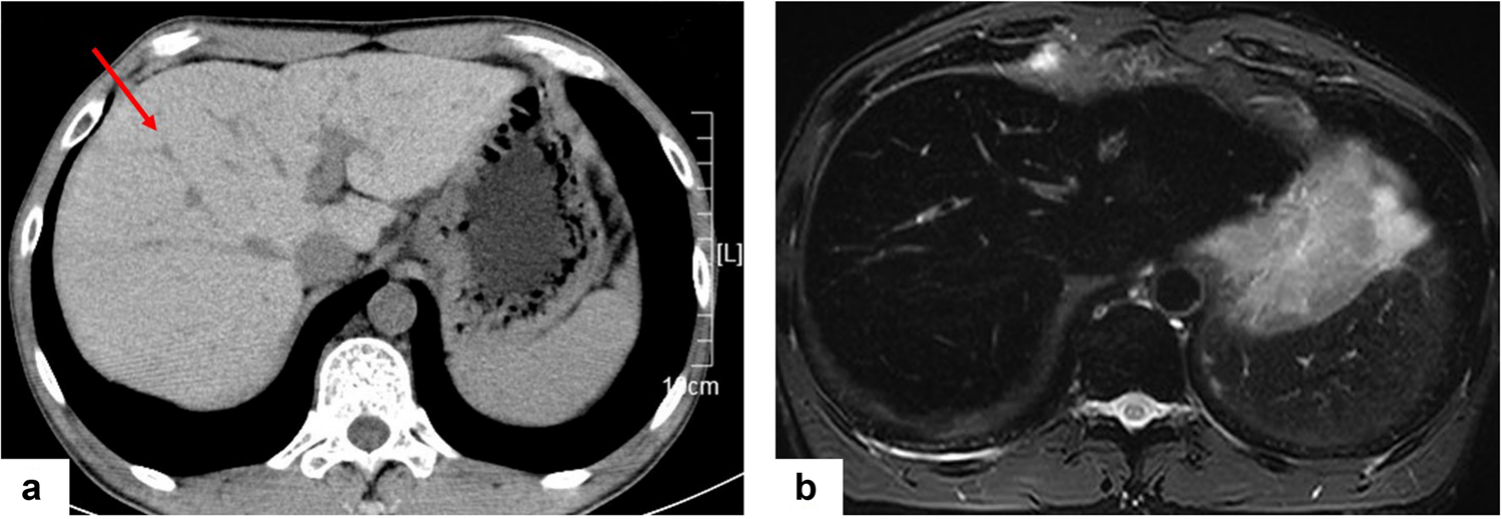
Imaging features of patients. (a) The density of the liver parenchyma increases diffusively in the CT portal vein stage, with a CT value of approximately 96 HU. Liver tissue sampling points (arrows). (b) In the magnetic resonance imaging T2 stage, the signals of the liver and spleen were low.

The third stage, from 2019 to 2020, involved the diagnosis and treatment of hemochromatosis. The patient complained that his skin turned black, ALT was maintained at the upper limit of 1–2 ULN, his serum iron level was 66.8 µmol/L (11.0–30.0 μmol/L), and ferritin was 7,532 μg/L (<200 μg/L). Further, enhanced computed tomography (CT) showed a large liver volume, a moderate proportion of liver lobes, small wavy change in the left edge of the liver lobe, diffuse increase in the density of liver parenchyma, and a CT value of approximately 96 HU (Figure 1a). In addition, magnetic resonance imaging showed low signals in both the liver and spleen (Figure 1b), indicating hemochromatosis.

**Figure 2:**
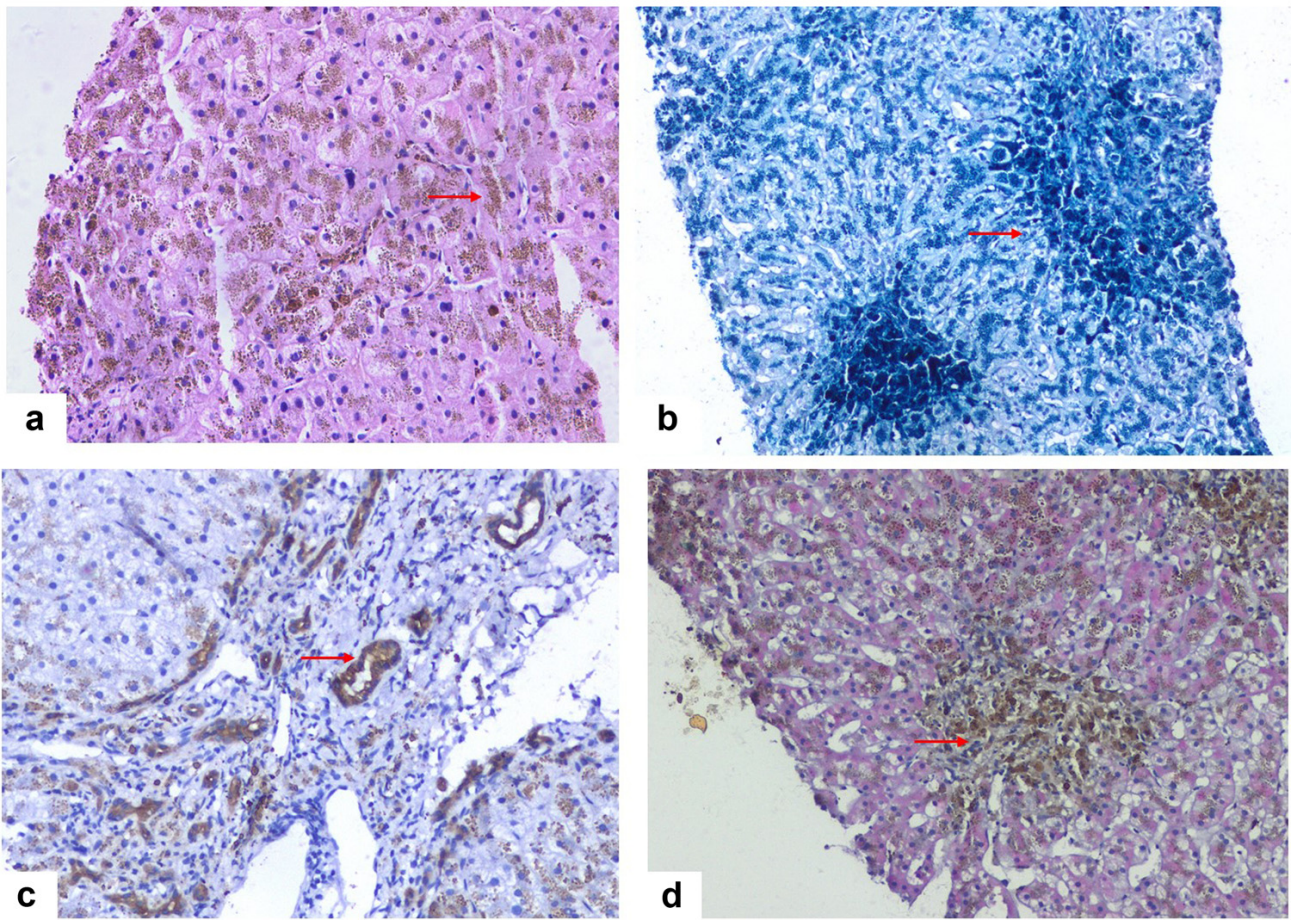
Pathological features of the liver tissue. (a) Hepatocytes filled with iron particles (arrow). Hematoxylin–eosin staining (10 × 20). (b) The portal area covered with blue iron particles (arrows). Prussian iron staining (10 × 20). (c) Immunohistochemical staining for cytokeratin 7 shows hyperplasia of the bile duct and deposition of iron particles in the bile duct (arrow; 10 × 20). (d) Deperiodic acid-Schiff staining shows iron particles in the macrophages (arrow; 10 × 20).


The pathological characteristics of the liver biopsy are shown in Figure 2. The liver cells and the epithelium of the small bile ducts in the submitted liver tissue were filled with iron particles, small bile ducts were proliferated, there was no obvious damage, no necrosis of liver cells was observed and no interfacial inflammation was noted. A small number of inflammatory cells infiltrated the portal tract, fibrous tissues in the portal tract proliferated and formed fibrous septa, no nodules were found, and the lobular structure was still clear. Special staining: Masson (+), Fe (++++), Cu (−), D-PAS (−); immunohistochemical labeling: HBsAg (−), HBcAg (−), CK19 (+), CK7 (+), CD138 (−), CD38 (−), IgG4 (−), CD10 (−). Hemochromatosis was considered, and gene detection of known mutation sites in hemochromatosis was negative for both the parents and patients. Point mutations and small deletion–insertion mutations in 20,858 gene exons and the adjacent ±20 bp region were detected by the second whole disease exon assay. If no mutation sites were found, primary hemochromatosis was excluded, or SH was diagnosed. Weekly bloodletting therapy (400 ml) and deferasirox were initiated. The fourth stage was the clinical cure of hepatitis B and the improvement of hemochromatosis. HBsAg was negative in February 2020. However, the liver function was always abnormal: ALT: 96 U/L, AST: 56 U/L, ALP: 136 U/L, GG: T87 U/L and HBV DNA: 3.12 × 10^3^ IU/L, considering occult hepatitis B and switching to tenofovir antiviral treatment. After 4 weeks, HBV DNA was undetectable (<10 IU/ml). One year after hemochromatosis treatment, ALT and AST levels were normal; however, ALP was still slightly elevated (<2 ULN). Tenofovir was discontinued after HBsAg was negative, and HBV DNA remained negative for 12 months. When ferritin was normal, and bloodletting was extended from once a week to 2–3 times a week, the patient’s skin returned to normal, liver function continued to be normal, and HBsAg was negative; however, anti-HBs did not appear (<2 IU/ml). At the time of writing of the article, the patient had only received deferasirox therapy without bloodletting treatment for 3 months, the patient refused a second liver biopsy, and the liver function and ferritin levels were normal.

**Ethics statement:** The ethics Statement was not applicable for case reports according to the Medical Ethics Committee of the Third Hospital of Zhenjiang Affiliated Jiangsu University; however, informed consent was obtained from the patient. This study was conducted in accordance with the principles of the Declaration of Helsinki.

## 3 Discussion

HH has been widely recognized, and the clinical manifestations of hemochromatosis-associated (HFE-associated) HH have been well studied in European populations [9,10]. In Asian countries, most HH cases are associated with non-HFE genes [11]. In addition, several novel gene variants associated with the regulation of iron homeostasis have been identified in Chinese patients with HH. In previous studies, SUGP2p.R639Q, BMP4p.R269Q, and DENND3p. L708V were first detected in patients with HH [12].

In this case, we diagnosed SH for the following reasons: (1) The patient had CHB in the past, and no symptoms or evidence of hemochromatosis was found. The patient had white skin, which turned dark in the previous 5 years and returned to normal with improved hemochromatosis. Ultrasound examination in the first and second stages of the disease did not reveal any changes in the iron deposition echo. (2) No HFE-related mutations were found in either gene. The patient and his parents were tested for the first time, and no HFE-related mutations were found. Second, no pathogenic gene mutations were detected using the whole-exon test. (3) After the clinical cure of hepatitis B, hemochromatosis improved.

Hemochromatosis may also be associated with HBV infection. First, in the 20-year history of CHB, the liver function ALT and AST levels were slightly abnormal for a long time (1–3 ULN). Although this was related to the long-term use of the second-line antiviral drug lamivudine and the addition of adefovir dipivoxil, the patient had a long-term complete virological response. The patient received antiviral treatment only after the liver enzyme levels became abnormal. Hemochromatosis is likely to exist, and doctors in the early stages have not diagnosed skin or other symptoms. Second, after hepatitis B was clinically cured, hemochromatosis improved.

In viral hepatitis, liver iron overload occurs in 35–56% of cases, especially in patients with chronic hepatitis C [13]. Giannini et al. studied 53 patients with chronic hepatitis C without HFE gene mutations and found that 19 (36%) had intrahepatic iron accumulation, which is related to liver inflammation and progressive cirrhosis [14]. In 81 male non-liver cirrhosis patients with HBV, 40% of the patients with CHB had elevated iron levels in the liver tissue, which was related to the severity of the corresponding liver disease [15], but not the HFE mutation. Higher serum iron levels are associated with poorer outcomes and prognoses in patients with CHB [8].

In an Iranian study, the frequency of the C282Y mutation associated with hemochromatosis was found to be significantly higher in 75 patients with CHB than in 194 patients without hepatitis B (4 vs 0%) [16]. The same phenomenon was also found in Taiwan, where the frequency of alleles for H1D mutations in hepatitis B-associated cirrhosis (63%) and hepatitis C-associated cirrhosis (6.9%) was higher than in healthy controls (2%) [17]. These mutations are considered risk factors for the progression of liver disease [18,19].

The degree and distribution of iron deposition in the liver tissue were evaluated using the Deugnier scoring system [20]. Iron deposition in the liver cells, liver sinuses, and portal areas in the three areas of the liver acinus was scored. Our patient’s iron deposition score was 30 points for liver cells in three zones (9 points in zone 1, 12 points in zone 2, and 9 points in zone 3), 10 points for the hepatic sinus (3 points for 1 zone, 4 points for 2 zones, and 3 points for 3 zones), and 9 points for the portal tract (3 points for connective tissue, 3 points for bile duct cells, and 3 points for the vascular wall), with a total score of 49 points.

In early HFE hemochromatosis, iron is located in the bile duct pole of hepatocytes, and the distribution of iron decreases from the sink area to the central lobular area, which is a typical pattern of parenchymal iron deposition. Histological characteristics are usually qualitative, and iron is often deposited in endothelial cells. Iron deposition in patients with hepatitis is observed in the liver cells, liver sinus cells, phagocytes, and endothelial cells in the portal area [21]. Although iron is an essential nutrient, it can damage cells. Hydroxyl radicals were produced during the formation of ferric iron from ferrous ions. The generated hydroxyl radicals destroy lipids, DNA, and proteins [22], and reactive oxygen species produced at high levels of cellular iron or hydroxyl radicals cause instability and decomposition of phospholipid membranes. This process leads to iron death, an iron-dependent cell death mechanism [23] that can attack liver cell components [24].

Increased serum iron levels are associated with a high prevalence of HBV infection [25]. In addition, the serum iron levels of persistent HBV carriers are significantly higher than those of patients with viral infections [26]. Serum iron and ferritin levels increased in patients with CHB. In other studies, serum transferrin level and total binding capacity decreased, and transferrin saturation increased [27,28]. Increased iron release from infected and damaged hepatocytes is speculated to be the cause of this situation. The success of lamivudine treatment is associated with a decrease in serum ferritin levels. Ohkoshi et al. have also shown that successful antiviral treatment resolved disease-related iron overload in these patients [29].

However, it remains controversial whether iron promotes or inhibits HBV replication. Iron overload in HepG2 cells can indirectly affect the HBV life cycle through cell cycle arrest and directly affect the HBV life cycle by inhibiting viral DNA secretion. They also indicated that careful evaluation of the iron depletion program for HBV-infected patients is required because reducing viral markers in the serum after iron depletion may not reflect a reduction in viral replication [30]. Viral DNA encodes its own ribonucleotide reductase to ensure nucleotide supply. If viral enzymes cannot obtain cellular iron owing to the use of cell-permeable ferrous chelating agents (such as 2,2′-bipyridine), viral replication is eliminated [31]. Hydroxyurea and deferoxamine may inhibit the production of viral particles via different mechanisms [30]. This mechanism may explain the disappearance of HBsAg and the low-level replication of HBV DNA in our patients. Some studies have shown that Fe promotes HBV replication [32,33,34]. The different effects of iron may depend on the different stages of liver disease in the included studies.

In conclusion, we reported a case of CHB complicated by hemochromatosis. The development of hemochromatosis is related to the repeated delay and non-recovery of CHB. Repeated necroinflammation in the liver cells causes liver iron overload. Although the application of tenofovir causes undetectable HBV DNA, the disappearance of HBsAg in the first place cannot be attributed to the effect of tenofovir.
